# The Role of Recipient Characteristics in Health Video Communication Outcomes: Scoping Review

**DOI:** 10.2196/30962

**Published:** 2021-12-30

**Authors:** Daniel Adrian Lungu, Jo Røislien, Siri Wiig, Marie Therese Shortt, Francesca Ferrè, Siv Hilde Berg, Henriette Thune, Kolbjørn Kallesten Brønnick

**Affiliations:** 1 SHARE – Centre for Resilience in Healthcare, Department of Quality and Health Technology Faculty of Health Sciences University of Stavanger Stavanger Norway; 2 Management and Health Laboratory Institute of Management Scuola Superiore Sant'Anna Pisa Italy

**Keywords:** health communication, video communication, communication outcomes, recipient characteristics, recipient factors, health video communication

## Abstract

**Background:**

The importance of effective communication during public health emergencies has been highlighted by the World Health Organization, and it has published guidelines for effective communication in such situations. With video being a popular medium, video communication has been a growing area of study over the past decades and is increasingly used across different sectors and disciplines, including health. Health-related video communication gained momentum during the SARS-CoV-2 pandemic, and video was among the most frequently used modes of communication worldwide. However, although much research has been done regarding different characteristics of video content (the message) and its delivery (the messenger), there is a lack of knowledge about the role played by the characteristics of the recipients for the creation of effective communication.

**Objective:**

The aim of this review is to identify how health video communication outcomes are shaped by recipient characteristics, as such characteristics might affect the effectiveness of communication. The main research question of the study is as follows: do the characteristics of the recipients of health videos affect the outcomes of the communication?

**Methods:**

A scoping review describing the existing knowledge within the field was conducted. We searched for literature in 3 databases (PubMed, Scopus, and Embase) and defined eligibility criteria based on the relevance to the research question. Recipient characteristics and health video communication outcomes were identified and classified.

**Results:**

Of the 1040 documents initially identified, 128 (12.31%) met the criteria for full-text assessment, and 39 (3.75%) met the inclusion criteria. The included studies reported 56 recipient characteristics and 42 communication outcomes. The reported associations between characteristics and outcomes were identified, and the potential research opportunities were discussed. Contributions were made to theory development by amending the existing framework of the Integrated-Change model, which is an integrated model of motivational and behavioral change.

**Conclusions:**

Although several recipient characteristics and health video communication outcomes were identified, there is a lack of robust empirical evidence on the association between them. Further research is needed to understand how the preceding characteristics of the recipients might affect the various outcomes of health video communication.

## Introduction

### Communication in Public Health Emergencies

Effective communication in public health emergencies is crucial, as people need to not only know but hopefully also understand the health risks they face and what actions they can take to protect themselves, their close ones, and the society from health hazards. The importance of communication in public health emergencies has been highlighted by the World Health Organization in the guidelines for risk communication in public health emergencies policy and practice [1]. The guidelines reflect the complexity of the topic, as several dimensions prove to determine the effectiveness of communication and must therefore be taken into consideration. Dimensions include information accuracy, timeliness and frequency of communication, clarity of language, use of appropriate media channels, building trust with local communities, the use of visuals in combination with—or instead of—text, and the use of new communication channels such as social media. Adding to the complexity, multimedia approaches have been found to be more effective than single media approaches [2].

In recent years, video communication has received increased attention across multiple fields, from education to science, risk, and health communication. Video allows rapid communication, is flexible and able to incorporate empathy, and has good outreach potential. Video communication gained further momentum during the COVID-19 pandemic, and most of the communication aimed at the population was through video. The effectiveness of delivering education through video has been widely investigated even before 2019 [3-7]; however, the COVID-19 pandemic has magnified and speeded up its adoption, and consequently, we can see increased research efforts in the area of effective communication using video [8-14].

### Health Communication

Health communication, which is defined as the dissemination and interpretation of health-related messages [15], is a well-established research area, with >300,000 search results in Google Scholar. A total of 4 elements have emerged from this extensive body of literature. The first element is the pervasive effort of theorizing health communication and putting it into practice [16-21]. The second element is the importance of cultural context for the planning and effectiveness of health communication interventions. On the one hand, different cultures require different health communication strategies [22,23]; however, within the same cultural context, different population groups need diversified communications to ensure effectiveness [24-26]. Thus, international literature often mentions the terms *tailored* and *targeted* health communication strategies designed to enhance the relevance of health information to a given audience [27,28]. The third element is empirical evidence concerning the effectiveness of health communication in achieving behavioral change or other public health goals (eg, the eradication of polio and other immunization coverage interventions) [29-32]. The fourth element is the challenge of reshaping and adapting health communication to reflect technological developments and societal changes. Indeed, the web-based environment, social media, advancements in artificial intelligence, and developments of health care (eg, eHealth and telemedicine) pose a challenge to health communication in the information age [33-37]. Furthermore, societies—especially those with universal health care systems—are called to tackle the health inequalities arising from technological advancements. Health communication must consider the digital divide and the different digital health literacies of the population to be effective [38]. This can be extremely relevant during pandemics, as, in the case of the COVID-19 pandemic, the groups affected by the digital divide and generally having less digital health literacy were the most affected [39-41]. ‬‬‬‬‬‬‬‬‬‬‬‬‬‬‬‬‬‬‬‬‬‬‬‬‬‬‬‬‬‬‬‬‬‬‬‬‬‬‬‬

Although much research has been conducted on video communication itself, there is less evidence regarding whether the outcomes (eg, knowledge, attitudes, compliance, and behavior) of health video communication are affected by the various characteristics (eg, sociodemographic, personality and values, and environmental factors) of the *recipients* of this communication. Health video communication refers to communicating health information through video format regardless of style (eg, animation, text over image, interviews, and voice over image). Health communication is multifaceted and variably affected by the messenger, message attributes, and recipient characteristics (eg, sociodemographic, personality and values, and environmental factors). This study aims to address this gap by identifying the relevant recipient characteristics and health video communication outcomes reported in the literature and the relationship between them. In this review, the concept of health video communication does not refer solely to public health communication videos but rather to health videos in general, including instructional videos for patient training and education, videos from public health prevention campaigns, and in-hospital informational videos.

### A Health Communication Framework

**Figure 1:**
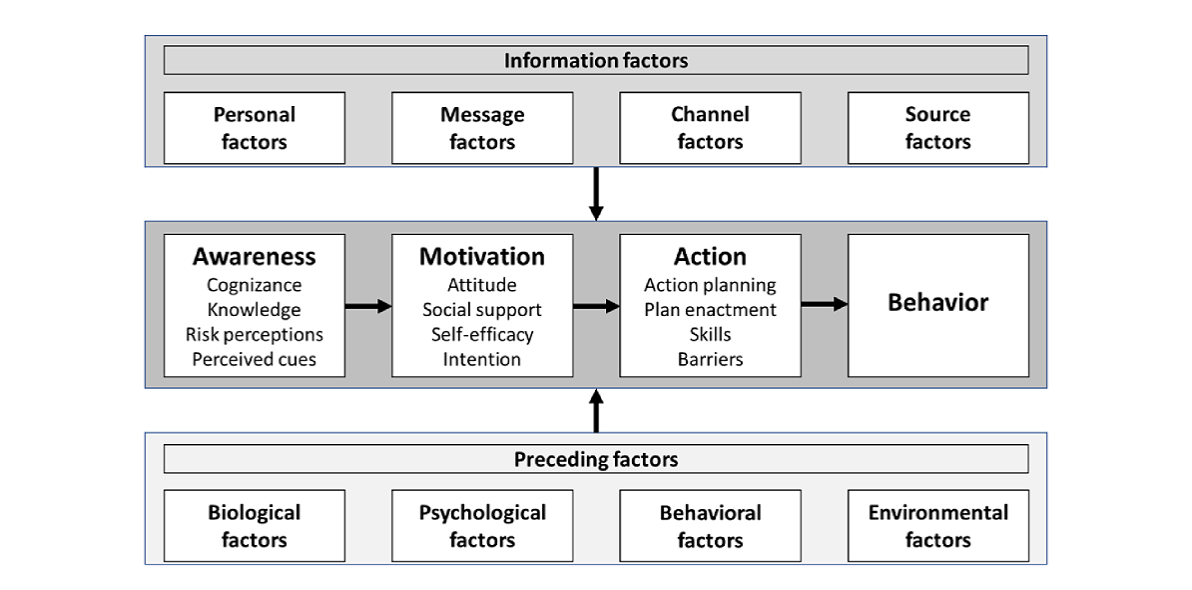
The Integrated-Change model.

For this study, we chose the Integrated-Change (I-Change) model (Figure 1) developed by Hein de Vries [42] as the study’s theoretical framework. As the driving research question is to identify recipient characteristics, outcomes of health video communication, and any relationship between them, the I-Change model fits the research question and the methodological approach of this study. Moreover, the categorization presented in the model is useful for discussing results and understanding the current state of knowledge and research practice. The I-Change model is an integrated model of motivational and behavioral change that combines elements from the theory of planned behavior [43], the social cognitive theory [44], the Transtheoretical Model of Health Behavior Change [45], the Health Belief Model [46,47], and the goal-setting theory [48,49]. The integration of these elements into a single health communication framework has made the adoption of the I-Change model appropriate as a basis for this study. The model states that both covert (hidden and not observable) and overt (visible and observable) behavior is determined by a person’s motivation or intention. In turn, motivation depends on attitudes, social influences, and self-efficacy. Attitudes are defined as the perceived cognitive and emotional advantages and disadvantages of a person’s behavior. A person’s social influences comprise social modeling (the perception of others having this behavior), social norms (the norms that people have regarding this behavior), and social support received from others when performing the behavior. Self-efficacy refers to the personal judgment of how well one can execute the courses of action to deal with prospective situations [44].

The model assumes that the communication outcomes (awareness, motivation, action, and behavior) depend on 2 determinants: information factors and preceding factors.

The information factors (Figure 1) have long been debated in international literature, and the importance of the source, channel, message, and messenger’s personal factors in influencing the outcomes of communication has been demonstrated in a wide range of disciplines, including, but not limited to, health, education, marketing, and risk communication [23,50-54].

The preceding factors (Figure 1) are predisposing factors of the recipients of the communication and comprise four categories: biological factors (eg, gender or sex and ethnicity), psychological factors (eg, personality, depression, and anxiety), behavioral factors (eg, lifestyle and adherence to recommendations), and environmental factors (eg, public health policies and availability or lack of resources at the community level). We are aware that gender is not a biological factor and is distinct from sex, but for the purpose of this study, we chose not to further elaborate on the difference between the 2 concepts. The terminology introduced by the I-Change model was adopted, and throughout the article, the terms *preceding factors* and *characteristics* of recipients are used interchangeably. This study focuses on the influence of preceding factors on health video communication outcomes.

## Methods

### Overview

The literature on recipient characteristics is heterogenous with regard to subject groups, methods, research questions, and disciplines. Thus, a scoping review approach was chosen to identify the nature and degree of evidence available in the international literature. Our review followed the five stages according to the methodological framework developed by Arksey and O’Malley [55], which we chose for its completeness: (1) identification of the research questions, (2) identification of relevant studies, (3) selection of studies, (4) data extraction and management, and (5) summary and analysis of results.

### Research Questions

This scoping review aimed to explore the role that recipient characteristics (biological, psychological, behavioral, and environmental factors) play in the outcomes of health video communication by (1) identifying which recipient characteristics and outcomes of health video communication were reported in peer-reviewed literature, (2) adapting the I-Change model to health video communication [42], (3) providing a categorization of both recipient characteristics and outcomes into the adapted model, and (4) investigating the relationships between recipient characteristics and health video communication outcomes reported in the international literature. This process will contribute to the existing knowledge on health communication by providing a comprehensive framework for health video communication effectiveness studies. The research questions driving this study were as follows:

1. What are the characteristics of recipients that might influence the outcomes of health video communication?
2. What are the outcomes of health video communication?
3. What relationships exist between the recipient characteristics and the outcomes of health video communication?

### Literature Identification

We searched three main databases for public health, social sciences, and biomedical studies on health video communication: PubMed, Scopus, and Embase. Additional literature was identified using the snowball method [56,57]. The relatively large number of papers identified in the initial search led us to limit the search to peer-reviewed literature. The search string was designed by relying on the population-concept-context framework, as suggested by the scoping reviews chapter of the Joanna Briggs Institute Manual for Evidence Synthesis [58]. The framework comprises defining a string for each component and subsequently combining them into the final search string. The string for the population component was (people OR patient* OR recipient* OR receiver* OR viewer* OR person).

The string referring to the concept was ([past OR previous OR prior] AND [knowledge OR experience]) OR ([person* OR individual*] AND [characteristic* OR propert* OR value*]) AND (outcome* OR attitude* OR behavi* OR accept* OR learn* OR react* OR respon*).

The string for the context component was (communic* AND video AND health).

Data from the 3 databases were extracted on December 12, 2020. The search was limited to results published in English only, whereas no restriction was applied to the publication year. The complete search strategy was validated by 2 of the coauthors and is available in Multimedia Appendix 1.

### Selection of Studies

The first step of the selection process comprised removing duplicates (approximately one-third) to confirm the completeness of the search string and strategy. In addition to incomplete or unavailable studies, studies were excluded based on title and abstract assessment if (1) they did not focus on recipient characteristics or on specific populations (eg, people who are visually impaired or deaf), (2) they did not focus specifically on health video communication (video used for other purposes, eg, video as a recording tool, referred to video games, video teleconference, video for the education of medical students, video calls, video physician–patient communication, or video simulation), or (3) they did not use video as the main communication method. Studies were included if they met both of the following criteria: (1) they focused on health video communication, and (2) they took into account at least one recipient characteristic. No inclusion or exclusion criteria were set for the design of the identified studies. Studies included in this phase went through full-text assessment, and the final inclusion depended on their coherence with respect to the 2 inclusion criteria mentioned above. The criteria and the results of each stage were made available to all the coauthors, and their feedback was used to solve potential inconsistencies among the scope of the study, the inclusion and exclusion criteria, and the selected articles.

### Data Extraction

Microsoft Excel was used to create data extraction forms. For title and abstract assessment, the information stored were the digital object identifier, inclusion or exclusion status, and reason for exclusion. In the second stage of full-text assessment, the information about the publication year, journal, authors, title, document type, country, research domain, recipient characteristics, and reported outcomes were added. All the selected papers were saved using Mendeley, and the library was shared among all the coauthors.

### Data Analysis

The extracted data were imported into the NVivo (version 12 Pro; QSR International). The relevant topics, both regarding the recipient characteristics and communication outcomes, were coded. A nested coding methodology [59] was used to organize the information in layers. The relationships between nodes were also coded to keep track of the associations between recipient characteristics and communication outcomes reported in the literature. The found relationships are reported in the description of outcomes in the *Results* section.

After the completion of the coding process, we moved on to define a conceptual framework, starting with the I-Change model and the way it highlights how the 4 dimensions of health communication outcome (awareness, motivation, action, and behavior) are influenced by information factors (eg, message, channel, and source) and preceding factors (biological, psychological, behavioral, and environmental factors). The existence of prior research allowed us to conduct a directed content analysis aimed at validating or conceptually extending the existing theoretical framework, as suggested by Hsieh and Shannon [60]. The content analysis, and especially the identification of key concepts or variables as initial coding categories, was guided by a structured and deductive approach according to the I-Change model [60-62].

The need to adapt the original model emerged at the first attempt to allocate the coded nodes to model categories, as some nodes lacked a fitting category in the original I-Change model. A similar approach of using content analysis to conduct knowledge building and theory development was described by Finfgeld-Connett [63]. The model was refined by an initial discussion between 2 coauthors (DAL and KKB) and then validated by the whole research group.

The final stage of data analysis was to assign nodes to the categories of the refined model. This process was conducted independently by 2 of the coauthors (KKB and DAL), and the divergencies were solved through discussion. In case the discussion did not lead to consensus, a third coauthor (SHB) was involved to have a majority. Finally, the relationships between the nodes reported in the literature were charted.

## Results

### Search Findings

**Figure 2:**
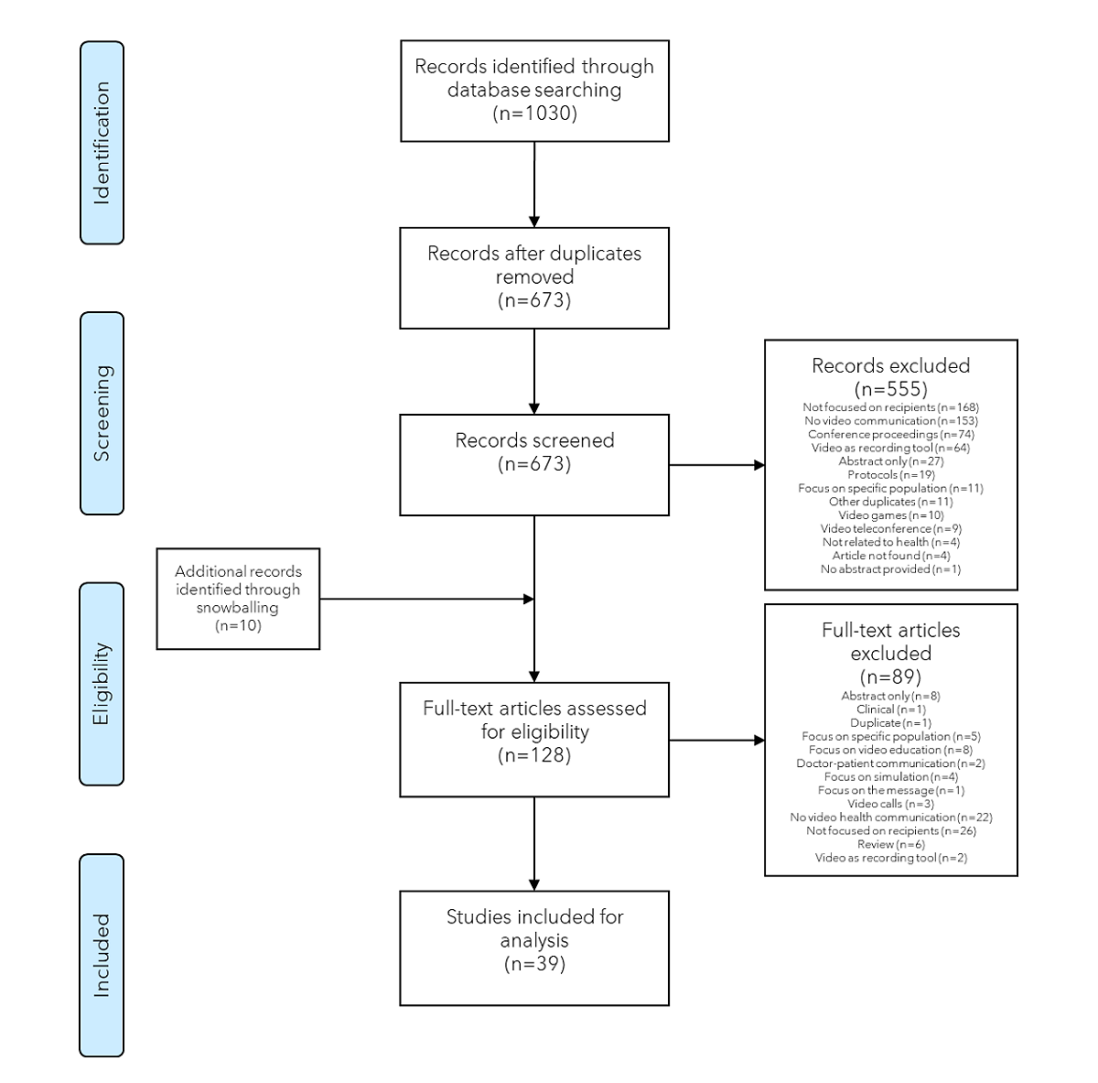
PRISMA (Preferred Reporting Items for Systematic Reviews and Meta-Analyses) chart of the selection process.

The database search strategy yielded 1040 records (331/1030, 32.13% from Scopus; 392/1030, 38.05% from PubMed; and 307/1030, 29.81% from the Embase). Approximately 34.66% (357/1030) were duplicates and thus removed, and the title and abstract assessment was performed for the remaining 65.34% (673/1030) of articles. Of the 673 articles, 555 (82.5%) were removed according to the eligibility criteria. A total of 10 additional documents were identified through cross-checking the references. In total, 128 documents were assessed for full-text. Of the 128 papers, 39 (30.5%) met the inclusion criteria and were included in the content analysis. The PRISMA (Preferred Reporting Items for Systematic Reviews and Meta-Analyses) chart of the selection process is presented in Figure 2. The characteristics of the selected studies are available in Multimedia Appendix 2 [64-100].

**Figure 3:**
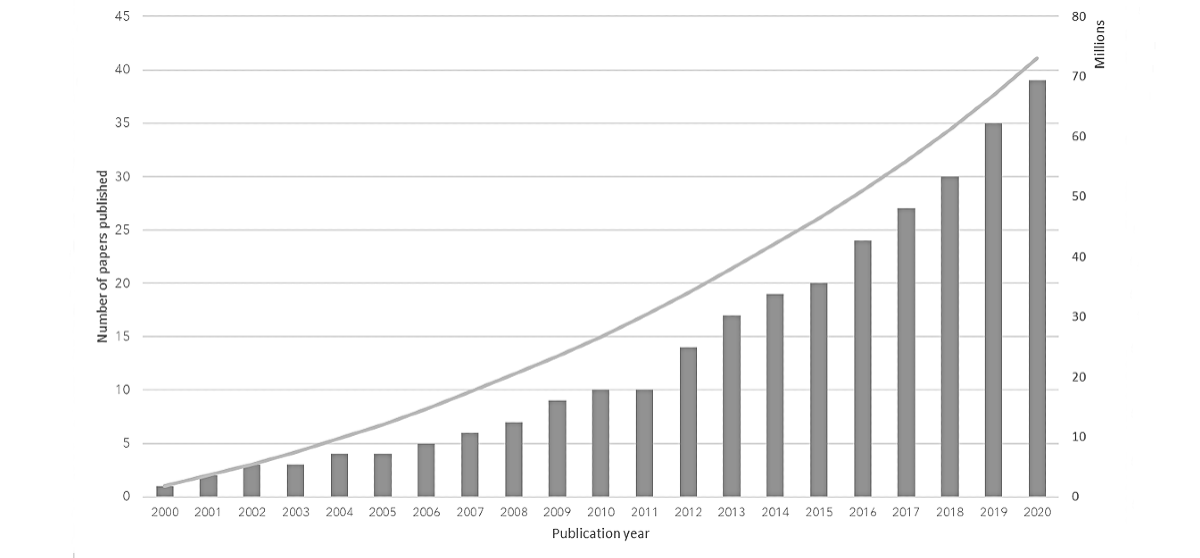
Cumulative number of papers published (worldwide and included in this study). Note that the x- and y-axes have different scales.

All the included studies were published between 2000 and 2020. There is an increasing worldwide publication trend over the past years. As highlighted in Figure 3, the publication trend for the articles included in this study is in line with the international trend, which displays the novelty and growing interest in this research topic.

### Recipient Characteristics

The included studies reported 56 different recipient characteristics. Some characteristics were frequently reported, with age (reported in 33/39, 85% of analyzed articles), ethnicity or race (26/39, 67%), gender or sex (25/39, 64%), and education (24/39, 62%) being the most investigated, whereas others such as sexual orientation, social network, emotional factors, or decisional control preferences being reported only by 3% (1/39) of studies each. Some characteristics were reported by a fair number of studies, such as income or socioeconomic status (SES; 13/39, 33%), beliefs and attitudes (12/39, 31%), level of knowledge (11/39, 28%), disease severity (10/39, 26%), previous experience (10/39, 26%), and health literacy (7/39, 18%). A complete overview of all the characteristics and the number of reporting documents is available in Multimedia Appendix 3, Table S1.

### Preceding Factors

#### Overview

The analysis identified 56 different recipient characteristics. Of the 56 characteristics, 48 (86%) could be assigned to a corresponding category in the I-Change model. However, some factors could not be naturally fitted in the I-Change model and were assigned to a new category termed *Knowledge factors*. Refer the *Additional Category of Knowledge Factors* section for further discussion into this.

#### Biological Factors

The category of biological factors encompassed 14% (8/56) of the recipient characteristics, namely age, comorbidities, ethnicity or race, gender or sex, self-reported health status, disease severity, current symptoms, and clinical factors. Most of these characteristics were frequently reported by the analyzed studies, with age, ethnicity or race, and gender or sex being the most investigated by international literature. Clinical factors, current symptoms, and comorbidities were rarely investigated as preceding factors that influence health video communication outcomes.

#### Psychological Factors

Almost half of the identified factors (27/56, 48%) were attributed to the category of psychological factors such as anxiety, depression, decisional control preferences, influential personal factors, information priorities, preferences, values, expectations, psychological distress, beliefs, attitudes, emotional factors, ability to process information, motivation, perception of treatment efficacy, treatment concerns, self-efficacy, trust in information sources, confidence, empathy, trust in health care, health locus of control, regulatory focus, confidence, hesitancy, sexual orientation, hope, and personal relevance. Furthermore, 13% (7/56) factors that were attributed to other categories could also have been included in this category, as they also have psychological implications but were a better fit into other categories. For example, comorbidities, self-reported health, disease severity, current symptoms, and clinical factors were assigned to the biological factors category; risk estimation was attributed to the knowledge factors category; and social norms were assigned to the environmental factors category.

#### Behavioral Factors

Only 4% (2/56) of the recipient characteristics were ascribed to the category of behavioral factors: involvement and self-reported adherence.

#### Environmental Factors

The environmental factors category encompassed 20% (11/56) of the identified characteristics. The most recurrent ones were income and SES (reported by 13/39, 33% of the studies), marital status (7/39, 18%), and type of health insurance (6/39, 15%). The remaining factors, generally seldom reported, were the level of information and support that people can rely on, employment, financial comfort, living with someone, social network, social norms, culture, and geographical location.

#### Additional Category of Knowledge Factors

The added category of knowledge factors contained 14% (8/56) of the factors. Similar to the biological factors, a significant degree of within-group variation was observed for these factors. Of the 39 included studies, the education level, knowledge, and previous experience were reported by 24 (62%), 11 (28%), and 10 (26%) studies, respectively. On the other hand, information, perceived knowledge, and health history were reported only by 3% (1/39) of the studies analyzed. The category also included 2 relevant factors that received an average amount of coverage in the international literature: health literacy and risk estimation and perception.

### Health Video Communication Outcomes

#### Overview

The included studies reported 42 different health video communication outcomes that covered a wide range of topics. The most frequently reported outcomes were knowledge, including an increase in knowledge and knowledge transfer (15/39, 38%), attitudes (9/39, 23%), behavior (9/39, 23%), and intentions (8/39, 21%). The least frequently included outcomes were awareness, compliance, activation, and attention, reported by only 3% (1/39) of studies each. Some of the outcomes that were reported by a fair number of studies included acceptance (7/39, 18%), beliefs (5/39, 13%), usefulness of the communication (4/39, 10%), and choice of treatment (4/39, 10%). A complete overview of all the health video communication outcomes is available in Multimedia Appendix 3, Table S2.

The 42 health video communication outcomes reported by the included studies were attributed to the categories of the I-Change model as follows: 28 (66%) outcomes were attributed to awareness and motivation (14/42, 33% of outcomes to each), 5 (12%) outcomes were attributed to a new category termed emotions, and the remaining 9 (21%) outcomes were attributed to the category of action that encompassed the original categories of action and behavior. These decisions will be further discussed in *The Revised I-Change Model* section.

#### Awareness

The most frequently reported outcomes belonged to the awareness category, which encompassed 33% (14/42) of the identified outcomes. Most of the included studies focused on elements of the awareness category as the main outcome of health video communication. The most frequently reported outcome was knowledge, which was explored in 38% (15/39) of the studies. The other included outcomes were expectations, usefulness, recall of information, uncertainty, comprehension, beliefs, perceived benefit, information, information processing, information seeking, perceived risk, perceived prevalence, and awareness.

Most of the reported evidence on awareness was focused on beliefs, knowledge gain, risk and prevalence perception, recall of information, and usefulness of communication. Syrjala et al [64] studied patient training in cancer pain management using integrated print and video materials and found that participants of color were much more likely to report the perception of severe pain than White participants (odds ratio [OR] 18.4, 95% CI 2.1-56.3; *P*=.01). In a study that assessed the effectiveness of smoking cessation communication on YouTube, Romer et al [65] found that ethnicity and current behavior affect beliefs: Hispanic participants were found to have stronger beliefs regarding the mortality effects of smoking, whereas current tobacco use was negatively related to these beliefs. Pretest beliefs, in turn, affected information processing, which is a relevant outcome in health communication [101]. In clinical trials video communication, Curbow et al [66] have found that higher levels of information processing were associated with more negative pretest beliefs about clinical trials. Recall of information was found to be correlated with knowledge factors, namely health literacy and level of education. In an oncology communication setting, Visser et al [67] showed that poor functional health literacy was a predictor for poor information recall when using a standard communication condition without emotional involvement. When the standard was replaced with emotion-oriented communication, health literacy did not have any statistically significant effect. In the context of maternal and neonatal health clips, results showed that the higher the level of education, the greater the information recall [68].

The generation and transfer of knowledge is one of the main health video communication outcomes investigated in international literature and reported by the studies included in this review [69-73]. Evidence regarding the impact of preceding factors on knowledge is mixed. McKenzie et al [74] reported no significant effect of health literacy and education on prepost maternal safety knowledge after exposure to injury prevention video recommendations. Similarly, Bekalu et al [75] investigated the effect of age, gender, education, race or ethnicity, and income on increase in the knowledge of pandemic influenza after video exposure and found no significant effect [75]. A similar result was obtained by Curbow et al [66] in the context of oncology clinical trial communication and by Phelan et al [76] in the context of back surgery, where a randomized trial showed no significant effect of preceding factors on knowledge increase [76]. Grindel et al [77] reported both a nonsignificant impact of preceding factors on breast cancer knowledge after video exposure and a significant drop in knowledge scores after 1 year, meaning that 1-time communication generally leads to increased knowledge in the short run; however, it may not be enough to sustain the knowledge gain in the long term. An opposite finding was reported by Hickey et al [78], who claimed to have achieved long-term (2-month follow-up) knowledge gain after a single viewing of a breast cancer video.

When analyzing the perceived smoking prevalence as an outcome of peer smoking cessation communication, Romer et al [65] found that Hispanic ethnicity (b=10.07; *P*=.02), female gender (b=2.67; *P*=.01), increasing age (b=1.55; *P*=.01), and current tobacco use (b=3.16; *P*<.001) were positively related to perceived smoking prevalence.

The final communication outcome belonging to the awareness category is perceived usefulness, and some studies have reported it to be relevant in the context of educational videos for patients with advanced gastrointestinal cancers [70] and in the context of urologic oncology of patients with localized prostate cancer [79]. However, the article by Albert et al [80] is the only study that investigated the relationship between preceding factors and perceived usefulness. Patients with higher self-confidence in using health devices and less severe conditions (*P*=.02) and patients of color (*P*=.03) perceived higher usefulness of telemonitoring devices. Higher perception of ease of use was affected by higher health literacy (*P*=.01), previous and current use history (*P*=.01), higher education level (*P*=.03), and married or cohabitating status (*P*=.02).

#### Motivation

The motivation category encompassed 33% (14/42) of the identified outcomes. Attitudes and intentions were the most investigated outcomes, being explored by 9 (23%) and 8 (21%) of the 39 analyzed studies, respectively. Acceptance, perceived self-confidence, perceived response efficacy, treatment preference, satisfaction, compliance, engagement, confidence, self-efficacy, and relevance are the remaining outcomes, and all of them were reported by less than 10% (4/42) of the selected studies.

More positive attitudes after exposure to video communication have been reported by the included studies in the context of outpatient surgical care and breast, prostate, and colorectal cancer [77,81,82]. Only Engler et al [81] reported that, in the German context, there were differences in the percentage of patients with positive attitudes toward health information on the web, with 92% of patients with colorectal cancer compared with 79% of patients with breast cancer and 53% of patients with prostate cancer.

The topic of confidence after exposure to health videos has been investigated in the United States regarding vaccinations, which is a topic that has been increasingly debated in the context of the COVID-19 pandemic. Nowak et al [83] found overall moderate confidence in the influenza vaccine (mean 2.78 on a scale of 1-5 scale, with *1* corresponding to *not at all confident*), with no differences between communication modalities (video, virtual reality, and electronic pamphlet story), although the influence of preceding factors was not investigated. When analyzing childhood vaccine-related confidence, Mendel-Van Alstyne et al [84] found that familiarity with the vaccine and high SES are associated with high confidence, whereas lacking information about issues that people were concerned about (eg, vaccine ingredients), uncertainty of the interaction of vaccines with children’s immune systems, and negative beliefs (eg, that vaccines can cause illnesses) led to low confidence.

Literature from health communication has investigated the effect of individual characteristics on intentions, focusing mainly on biological factors [102]. Results showed that high intentions toward activities that improve health are associated with being a woman [103-105], increasing age [106,107], and being White [108,109]. In the context of health video communication, all the included studies that focused on intentions either did not investigate the effect of preceding factors on them or reported a nonsignificant relationship [72,74,83,85,86].

#### Emotions

The added category of emotions received fair attention in previous studies, with 12% (5/42) of the identified outcomes belonging to the emotions category. The identified outcomes were emotional response, decisional conflict, decision quality, decision satisfaction, and reactions. Moreover, each emotional outcome was reported by a very limited number of studies, which highlighted the limited amount of focus these factors have attracted and a potential gap in the literature.

There is a general lack of evidence about how emotional factors affect communication outcomes. Although there is some evidence regarding emotional reactions and trust, nothing was reported regarding the acceptance of health messages or the effects on the decision-making process (eg, decision satisfaction and decisional conflict) of recipients in the studies that were eligible for inclusion in this review.

The emotional response to health video communication has been reported to be significantly influenced by the age and gender of the recipients. A study by Prieto-Pinto et al [68] found, by using pupillary dilatation measures, which correlates with autonomous nervous system arousal, that men tend to have a greater emotional reaction after being exposed to maternal and neonatal health video clips. In a study conducted in China that aimed to test video versus virtual reality health communications, Liu et al [87] found that the young and older population differ in positive emotions after being exposed to communication: the young individuals show a greater emotional response to virtual reality, whereas the older individuals show a greater emotional response to health video communication. Of the 39 included studies, trust, which is a key outcome of health communication, was rarely investigated, with only 3 (8%) of the papers included in this review reporting on trust and only 2 (5%) providing empirical evidence regarding the impact of prior mistrust, age, and education on trust. By using a multilevel analysis model in the context of oncology communication, Hillen et al [88] reported a positive effect of age (*P*<.001) and a negative effect of education (*P*=.04) on trust. By applying structural equation modeling to investigate the reactions to survivor of breast cancer stories, McQueen and Kreuter [89] found that the level of medical mistrust at baseline affects the evaluation of the communication video (*β*=−.29; *P*<.001). The lack of further empirical evidence on trust highlights a potential gap in the literature that future research should address.

#### Action

The action category, which was obtained by merging the categories of action and behavior of the original I-Change model, encompassed 21% (9/42) of the identified health video communication outcomes. Outcomes belonging to this category received little attention in the literature, with all of them reported by less than 10% (4/42) of the included studies. Capabilities, time to treatment, quality of communication, participation, activation, spreading the message, and behavior complete the list of outcomes included in the action category.

Adherence to treatment is one of the main action outcomes of health video communication. Although adherence was often reported by the studies included in this review, there is little evidence of how the preceding factors affect it. In a study that investigated the risk of nonadherence to antiplatelet medication at the time of coronary stent placement, Palacio et al [90] reported no significant effects of gender, race or ethnicity, health literacy, income, education, and access to care on adherence. Instead, adherence was statistically higher for patients with a spouse or a domestic partner (*P*=.05), with a low Charlson comorbidity score (*P*=.03) and high English proficiency (*P*=.05). Low adherence was observed among those with a positive screening for depression and among patients with moderate to severe depressive symptoms (*P*=.01).

In the context of maternal vaccine information communication in the United States, Dudley et al [86] investigated the factors associated with referring close contacts after exposure. The likelihood of referring contacts increased for women who intended to receive a maternal influenza vaccine (OR 1.37, 95% CI 1.04-1.81), whereas it decreased for those who were unsure about their infant vaccine intentions (OR 0.47, 95% CI 0.27-0.83). Participants were more likely to refer contacts if they were confident in the safety (OR 1.64, 95% CI 1.13-2.38) and efficacy of the maternal vaccine (OR 1.9, 95% CI 1.21-2.98), had higher perceived susceptibility to (OR 1.62, 95% CI 1.1-2.4) and severity of (OR 2.19, 95% CI 1.28-3.73) influenza during pregnancy, and had trust in the maternal vaccine information from academic institutions (OR 1.56, 95% CI 1.09-2.25) and the infant vaccine information from the Center for Disease Control (OR 1.44, 95% CI 1.02-2.05) and academic institutions (OR 1.85, 95% CI 1.27-2.71). The associations between the likelihood of referring contacts and ethnicity, education, state, or having prior children were not statistically significant.

None of the included studies reported any associations between the preceding factors and the other action outcomes such as activation, behavior, participation, time to treatment, or treatment choice.

Almost one-third (12/39, 31%) of the papers included in this analysis focused on both preceding factors and health video communication outcomes but did not investigate the relationship between them. Information about the preceding factors was collected and used mainly for descriptive purposes rather than explanatory variables [74,91-100,110].

It is noteworthy to observe how the action category received relatively low attention from the included studies, as health videos are often designed and used to drive behavior and behavioral change (eg, smoking cessation, vaccination decisions, and weight loss). Indeed, a tendency to focus on awareness and motivation rather than on the actual behavioral outcome of the recipients emerged from the included studies.

### The Revised I-Change Model

**Figure 4:**
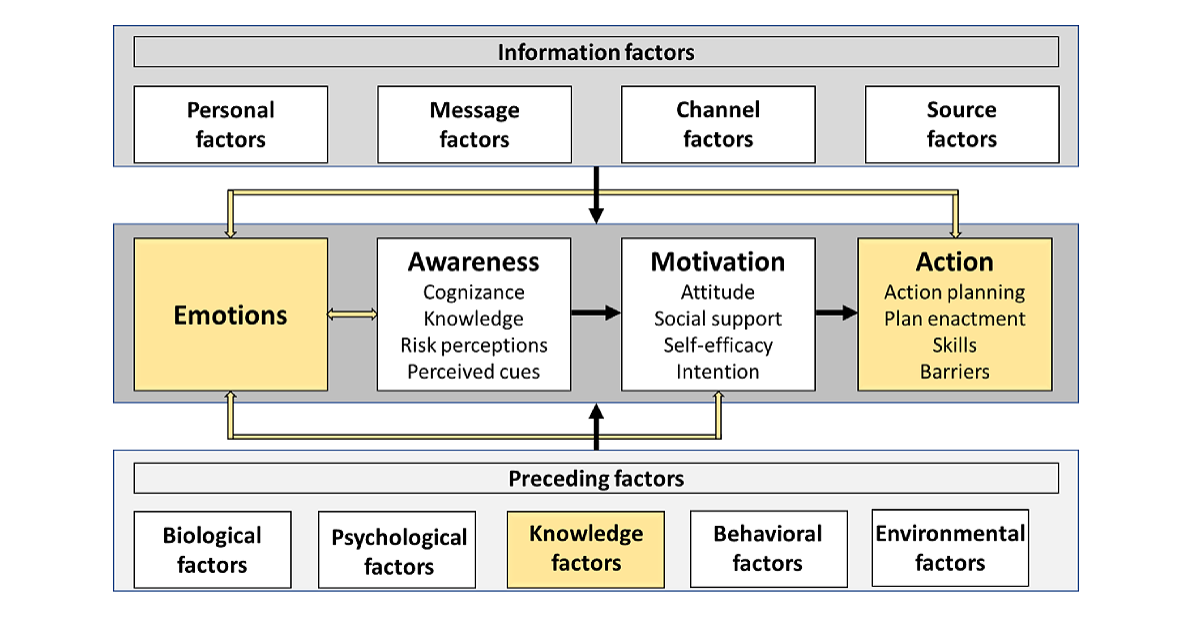
The revised Integrated-Change model.

When analyzing the included studies—coding the content and identifying relevant nodes regarding both recipient characteristics and communication outcomes—some gaps emerged in the I-Change model when applied to health video communication. Thus, we revised the I-Change model to adapt it to health video communication and obtained an adapted version (Figure 4) that fits the purpose and comprises three main novelties with respect to the original version:

- The inclusion of a fifth category termed knowledge factors within the preceding factors
- The merging of the action and behavior outcome categories into a single category termed action
- The inclusion of an emotions category among the outcomes

The category of knowledge factors was included as 1 of the preceding factors categories as many included studies reported multiple characteristics associated with knowledge that did not meaningfully belong to biological, psychological, behavioral, or environmental factors. Examples of such characteristics include the recipients’ level of knowledge, health literacy, level of information, ability to process information, and information priorities.

The original model foresees action and behavior as distinct communication outcomes, whereas our analysis demonstrated an overlap between the 2 categories using the data from the 39 studies included in this review. Thus, we decided to merge them into a single category termed as action.

The emotional dimension has been shown to play an important role in multiple outcomes of communication: risk perception, judgment, and decision-making [111]. Emotions can both provide significant inputs to judgments and decision-making and can fundamentally change the process of judging and deciding, especially when knowledge about the events is not easily remembered or expressed [112]. Moreover, not only are emotions influenced by preceding and information factors but are also in a mutual relationship with the other outcome categories, as highlighted by the 2-way arrows in Figure 4. This represents an important amendment to the original model, as it considers the feedback effect that awareness, motivation, and action have on emotions.

## Discussion

### Principal Findings

The included studies provide an extensive overview of the recipients’ preceding characteristics that might affect a wide range of health video communication outcomes and present evidence on the relationship between them. The trend observed in Figure 3 displays the novelty and growing interest in this research topic.

Although subsequent analysis revealed that many of the preceding factors belong to the psychological category, only a limited number of papers reported evidence of their impact on outcomes, inviting further research in the area. Biological factors were often reported by the analyzed studies.

Although knowledge and environmental factors received fair attention in international literature, systematic and robust evidence of their effect on communication outcomes is scarce. Moreover, the recipients’ first language and their ability to speak and understand the content of videos were surprisingly not identified as relevant factors. This is because the selected studies included only people who were able to speak the language presented in the communication. Further research investigating the magnitude of the impact of linguistic skills on outcomes would positively contribute to the effectiveness of health communication.

The category of behavioral factors appears to be underinvestigated, with only 2 factors identified in all 39 studies and no evidence available on the relationship with outcomes. Although the awareness and motivation categories included the most communication outcomes, those belonging to the action and emotions categories were less reported by the included studies, and there is very little evidence of how they are affected by the preceding factors. Communication and health research would benefit from further efforts focusing on the action category, as it currently represents less than 10% (4/42) of the outcomes identified in our study, with most studies preferring to focus on awareness and motivation outcomes. This number appears low with respect to the general aim of most health communication interventions aimed at changing behavior.

The scarcity of evidence regarding emotions is particularly relevant for effective health communication. Feelings and moods motivate people to reproduce those feelings and moods, whereas, in risk communication, the topic communicated (eg, earthquakes and other natural disasters) usually evokes negative feelings and moods, motivating people to act to avoid them. Emotions are states that are not under voluntary control but are shaped and learned associatively through experiences while being partly innate at birth. Emotions affect attention, memory, motivation, and action [113]; therefore, we included them as a category of preceding factors in the adapted version of the I-Change model.

Our contribution to theory development followed the research approach, in which the theory is understood as emerging from data [114,115]. Indeed, the empirical approach comprises the development of a new theory by relying on empirical observations followed by careful analysis and verification of hypotheses. We started with an existing theoretical framework; however, the empirical data highlighted the misfit of the I-Change model to the topic of health video communication. Therefore, through data analysis, we revised the original model and adapted it to the specific characteristics and needs of health video communication.

As we decided to conduct a scoping review, we are aware of the common limitations of this approach [116]. Information was gathered from a wide range of study designs and methodologies, and the quality of evidence was not formally evaluated. As for the results, it does not provide a synthesized answer to a specific question but rather an overview of the available evidence in the literature.

Further understanding of the gaps presented in this review could have a great impact on the effectiveness of public health emergency communication strategies, as in these contexts, the psychological and behavioral factors of people are key, and emotions are able to significantly affect their decisions and behavior.

### Conclusions

Although some evidence of associations between recipients’ preceding factors and health video communication has been reported in the literature, our analysis revealed a significant gap in the literature, with many health video communication outcomes and factors not yet explored. This scoping review of the available evidence demonstrated a potential research gap, especially concerning the emotional outcomes of communication and the behavioral and psychological preceding factors of recipients. The review showed that, currently, only a partial picture of the role of recipient characteristics on the outcomes of health video communication is available. Moreover, this study also contributed to the theoretical development of the I-Change model to adapt it to the needs of health video communication.

## Appendix

Multimedia Appendix 1 Complete search strategy and results.

Multimedia Appendix 2 Overview of included studies.

Multimedia Appendix 3 Overview of characteristics and outcomes.

## Abbreviations

I-Change: Integrated-Change
OR: odds ratio
PRISMA: Preferred Reporting Items for Systematic Reviews and Meta-Analyses
SES: socioeconomic status
