# Supplementary material for: The Role of Recipient Characteristics in Health Video Communication Outcomes: Scoping Review
Source: J Med Internet Res. 2021 Dec 30;23(12):e30962. doi: 10.2196/30962 (PMC8759013; doi:10.2196/30962)
Supplement: Multimedia Appendix 1 [file jmir_v23i12e30962_app1.docx]

**Appendix 1**

**Population =** (people OR patient* OR recipient* OR receiver* OR viewer* OR person)

**Concept =** ((past OR previous OR prior) AND (knowledge OR experience)) OR ((person* OR individual*) AND (characteristic* OR propert* OR value*))) AND (outcome* OR attitude* OR behavi* OR accept* OR learn* OR react* OR respon*)

**Context =** (communic* AND video AND health)

**Combined search string =** ((past OR previous OR prior) AND (knowledge OR experience)) OR ((person* OR individual*) AND (characteristic* OR propert* OR value*))) AND (outcome* OR attitude* OR behavi* OR accept* OR learn* OR react* OR respon*) AND (communic* AND video AND health) AND (people OR patient* OR recipient* OR receiver* OR viewer* OR person)

**Search date =** 24.11.2020

**Results**

|  | **Scopus** | **Pubmed** | **EMBASE** | **Total** |
| --- | --- | --- | --- | --- |
| Number of hits | 331 | 392 | 307 | 1030 |
