# Supplementary material for: The Role of Recipient Characteristics in Health Video Communication Outcomes: Scoping Review
Source: J Med Internet Res. 2021 Dec 30;23(12):e30962. doi: 10.2196/30962 (PMC8759013; doi:10.2196/30962)
Supplement: Multimedia Appendix 2 [file jmir_v23i12e30962_app2.docx]

**Appendix 2**

| **Author(s)** | **Year** | **Method** | **Journal** | **Title** | **Country** | **Number of participants** | **Domain** | **Recipients' characteristics** | **Outcomes** | **Relationship found in the study** |
| --- | --- | --- | --- | --- | --- | --- | --- | --- | --- | --- |
| Albert et al.  [81] | 2016 | Cross-sectional, multicenter, international design | Journal of Telemedicine and Telecare | Factors associated with telemonitoring use among patients with chronic heart failure | USA, Denmark | 206 | Heart failure | Ethnicity, marital status, health literacy, education, anxiety, depression, previous experience | Usefulness, satisfaction | Y |
| Bekalu et al.  [76] | 2018 | Single site RCT | Preventive Medicine | The relative persuasiveness of narrative versus non-narrative health messages in public health emergency communication: Evidence from a field experiment | USA | 627 | Public health emergency | Education, knowledge, income, SES, age, gender/sex, ethnicity | Perceived response efficacy, knowledge | Y |
| Berry et al.  [80] | 2013 | Multi-center randomized trial | Urologic Oncology: Seminars and Original Investigations | The Personal Patient Profile-Prostate decision support for men with localized prostate cancer: A multi-center randomized trial | USA | 494 | Prostate cancer | Influential personal factors, information priorities, information and support, anxiety, decisional control preferences, current symptoms, ethnicity, age | Time to treatment, acceptance, usefulness, treatment choice, decisional conflict | Y |
| Butalid et al.  [100] | 2012 | Mixed methods | BMC Family Practice | Patients’ views on changes in doctor-patient communication between 1982 and 2001: a mixed-methods study | Netherlands | 108 | Health communication | Self-rated health, gender/sex, age, ethnicity, employment, education | Quality of communication | N |
| Cox et al.  [70] | 2014 | Theoretical framework development | American Journal of Respiratory and Critical Care Medicine | A Universal Decision Support System. Addressing the Decision-Making Needs of Patients, Families, and Clinicians in the Setting of Critical Illness | USA | - | Critical illness | Values, health literacy, preferences, expectations, risk estimation, attributes, psychological distress | Decision quality, decisional conflict, treatment choice, knowledge, uncertainty, satisfaction, participation, comprehension, quality of communication | Y |
| Curbow et al.  [67] | 2007 | Single site factorial design experiment | Journal of Health Communication | The Role of Physician Characteristics in Clinical Trial Acceptance: Testing Pathways of Influence | USA | 262 | Oncology | Knowledge, beliefs and attitudes, risk estimation, ethnicity/race, emotional factors, motivation, information, ability to process information | Knowledge, information processing, beliefs, attitudes, acceptance, satisfaction | Y |
| Danila et al.  [72] | 2016 | International prospective, longitudinal, observational study | Contemporary Clinical Trials Communications | A multi-modal intervention for Activating Patients at Risk for Osteoporosis (APROPOS): Rationale, design, and uptake of online study intervention material | USA | 18 | Osteoporosis | Preferences, efficacy perception, education, age, ethnicity | Knowledge, activation | Y |
| Deyo et al.  [106] | 2000 | 2 sites RCT | Medical care | Involving Patients in Clinical Decisions: Impact of an Interactive Video Program on Use of Back Surgery | USA | 393 | Surgery | - | Treatment choice, satisfaction, compliance, symptom and functional outcomes | N |
| Dudley et al.  [95] | 2020 | Multisite randomized trial | Vaccine | Factors associated with referring close contacts to an app with individually tailored vaccine information | USA | 1095 | Vaccines | Risk estimation, trust in information sources, treatment concerns, social norms, knowledge, perception of knowledge, social network, ethnicity, self-efficacy, beliefs and attitudes, education, confidence | Spreading the message, intentions, decisions, beliefs, attitudes | Y |
| Engler et al.  [82] | 2016 | Mixed methods (log file analyses, survey data and thematic analysis of focus group discussions) | Patient Education and Counselling | Using others’ experiences. Cancer patients’ expectations and navigation of a website providing narratives on prostate, breast, and colorectal cancer | Germany | 60 | Oncology | Gender/sex, age | Attitudes | Y |
| Epstein et al.  [71] | 2015 | Qualitative thematic content analysis of participants’' responses in a randomized trial | Psychooncology | We have to discuss it’: cancer patients’ advance care planning impressions following educational information about cardiopulmonary resuscitation | USA | 54 | Oncology | Values, preferences, knowledge, beliefs and attitudes, health literacy | Usefulness, knowledge, information, acceptance | Y |
| Geary et al.  [107] | 2006 | Multi-national focus group discussions | Health Education & Behaviour | Personal involvement of young people in HIV prevention campaign messages: the role of message format, culture, and gender | Brazil, Kenya, Nepal, Senegal | 41 | HIV | Personal relevance, culture, gender/sex | Engagement | N |
| Grindel et al.  [78] | 2004 | Multisite repeated measures intervention | Oncology Nursing Forum | The Effect of Breast Cancer Screening Messages on Knowledge, Attitudes, Perceived Risk, and Mammography Screening of African American Women in the Rural South | USA | 450 | Breast cancer | Risk estimation, knowledge, beliefs and attitudes, marital status, insurance type, age, location, income, SES, education, health history | Perceived risk, participation, knowledge, attitudes | Y |
| Hannawa  [108] | 2012 | Single site RCT | Swiss Medical Weekly | “Explicitly implicit”: examining the importance of physician nonverbal involvement during error disclosures | USA | 318 | Error disclosures | Sex, ethnicity, religion, previous experience | Satisfaction | N |
| Hickey et al.  [79] | 2013 | Test-retest | Oncology Nursing Society | Breast Cancer Education for the Deaf Community in American Sign Language | USA | 122 | Breast cancer | Knowledge, insurance type, perception of knowledge, age, education | Knowledge, behavior | Y |
| Hillen et al.  [97] | 2014 | Experimental video-vignettes study | Patient Education and Counselling | Does source of patient recruitment affect the impact of communication on trust? | Netherlands | 344 | Oncology | Disease severity, anxiety, trust in health care, education, gender/sex, age, health locus of control | Trust | Y |
| Kempenich et al.  [83] | 2018 | Multisite RCT | Journal of Surgical Education | Video-Based Patient Education Improves Patient Attitudes Toward Resident Participation in Outpatient Surgical Care | USA | 383 | Surgery | Previous experience | Attitudes | Y |
| Liu et al.  [96] | 2020 | Single site pre-post between-subject laboratory experiment | Cyberpsychology, Behaviour and Social Networking | The Effects of Viewing an Uplifting 360-Degree Video on Emotional Well-Being Among Elderly Adults and College Students Under Immersive Virtual Reality and Smartphone Conditions | China | 58 | Health communication | Age | Emotions | Y |
| Lutfey et al.  [109] | 2012 | Multisite factorial experiment | Health Services Research | Physician Styles of Patient Management as a Potential Source of Disparities: Cluster Analysis from a Factorial Experiment | USA | 256 | Cardiology | Income, SES, gender/sex, ethnicity, age | Decision making | N |
| McKenzie et al.  [75] | 2019 | National randomized controlled experiment | Journal of Health Communication | Maternal Knowledge, Attitudes, and Behavioural Intention after Exposure to Injury Prevention Recommendations in the News Media | USA | 1081 | Maternal injury prevention | Ethnicity, marital status, health literacy, education, income, age | Knowledge, intention, attitudes | N |
| McQueen and Kreuter  [98] | 2010 | Structural equation modelling | Patient Education and Counselling | Women’s cognitive and affective reactions to breast cancer survivor stories: A structural equation analysis | USA | 489 | Breast cancer | Previous experience, trust in health care, education, income, SES, disease severity, gender/sex, age, ethnicity, beliefs, and attitudes | Reactions, engagement | Y |
| Mendel-Van Alstyne et al.  [85] | 2018 | Multisite stratified moderator-lead focus groups | Vaccine | What is ‘confidence’ and what could affect it?: A qualitative study of mothers who are hesitant about vaccines | USA | 61 | Vaccines | Trust in information sources, insurance type, income, hesitancy, previous experience, expectations, beliefs and attitudes, education, ethnicity, age, knowledge, confidence | Acceptance, trust, confidence, adherence | Y |
| Morrongiello et al.  [110] | 2009 | Focus groups with randomly selected participants | Social Science and Medicine | Video messaging: what works to persuade mothers to supervise young children more closely in order to reduce injury risk? | Canada | 17 | Maternal injury prevention | SES, income, ethnicity, age | Reactions | N |
| Ng et al.  [111] | 2019 | Thematic analysis for developing a causal chain model | BMC Psychiatry | The mechanisms and processes of connection: developing a causal chain model capturing impacts of receiving recorded mental health recovery narratives | England | 40 | Mental health | Values, personality factors, preferences, sexual orientation, marital status, education, hope, disease severity, gender/sex, ethnicity, clinical factors, age, beliefs, and attitudes | Mechanisms of effects on recipients | N |
| Nowak et al.  [84] | 2020 | One-way between-subjects experimental design | Vaccine | Using immersive virtual reality to improve the beliefs and intentions of influenza vaccine avoidant 18-to-49-year-olds: Considerations, effects, and lessons learned | USA | 171 | Vaccines | Previous experience, gender/sex, confidence, education, beliefs and attitudes, knowledge, age | Intention, confidence, beliefs | Y |
| Otto-Salaj et al.  [94] | 2009 | Interview analysis, cluster analysis | The Journal of Sex Research | Reactions of Heterosexual African American Men to Women’s Condom Negotiation Strategies | USA | 172 | Sexual health | Previous experience, marital status, education, income, disease severity, ethnicity, beliefs and attitudes, knowledge, gender/sex, age | Behavior, intentions | Y |
| Palacio et al.  [99] | 2017 | Single site randomized clinical trial | Journal of Cardiovascular Nursing | Can We Identify Minority Patients at Risk of Non-Adherence to Antiplatelet Medication at the time of Coronary Stent Placement? | USA | 452 | Cardiovascular surgery | Disease severity, depression, trust in health care, ethnicity, self-reported adherence, marital status, education, insurance type, gender/sex, beliefs and attitudes, income, SES, health literacy, age | Adherence | Y |
| Perez et el.  [103] | 2016 | Mixed methods | BMC Medical Informatics and Decision Making | Characterizing internet health information seeking strategies by socioeconomic status: a mixed methods approach | USA | 78 | Health decision making | SES, education, income, health literacy, previous experience, insurance type, ethnicity | Information, information seeking, decisions | N |
| Phelan et al.  [77] | 2001 | Single site randomized trial | Spine | Helping Patients Decide About Back Surgery: A Randomized Trial of an Interactive Video Program | USA | 100 | Surgery | Knowledge, preferences, education, disease severity, gender/sex, age | Knowledge, treatment preference, decisions, reactions | Y |
| Prieto-Pinto et al.  [69] | 2019 | Randomized crossover trial | PLoS One | Effectiveness assessment of maternal and neonatal health video clips in knowledge transfer using neuromarketing tools: A randomized crossover trial | Colombia | 155 | Maternal care | Education, gender/sex, age | Emotions, recall of information, knowledge transfer | Y |
| Romer et al.  [65] | 2017 | Online RCT | Journal of Health Communication | Counteracting the Influence of Peer Smoking on YouTube | USA | 999 | Smoking | Previous experience, gender/sex, ethnicity, age | Beliefs, attitudes, perception of the prevalence | Y |
| Sacks et al.  [74] | 2013 | Multisite experimental design | Journal of Cancer Education | Testicular Cancer Knowledge among Deaf and Hearing Men | USA | 175 | Testicular cancer | Education, age, ethnicity | Knowledge | Y |
| Shi et al.  [101] | 2017 | Imaging genetics | Biological Psychology | Individual differences in the processing of smoking-cessation video messages: An imaging genetics study | USA | 53 | Smoking | Education, ethnicity, age, gender/sex | Behavior | N |
| Shi et al.  [102] | 2019 | Multisensory processing RCT | Psychology Research and Behaviour Management | Connectivity between visual and auditory cortices mediates the influence of argument strength on the effectiveness of smoking-cessation videos among smokers with low in sensation seeking | USA | 66 | Smoking | Gender/sex, age, ethnicity | Behavior | N |
| Syrjala et al.  [64] | 2008 | Multisite RCT | The Journal of the International Association for the Study of Pain | Patient training in cancer pain management using integrated print and video materials: A multisite randomized controlled trial | USA | 93 | Oncology | Disease severity, education, gender/sex, ethnicity, current symptoms, beliefs and attitudes, age | Beliefs | Y |
| Van Het Schip et al.  [104] | 2020 | Mixed methods | Journal of Medical Internet Research | Spoken Animated Self-Management Video Messages Aimed at Improving Physical Activity in People with Type 2 Diabetes: Development and Interview Study | Netherlands | 23 | Type 2 diabetes | Education, income, SES, gender/sex, health literacy, age | Behavior, awareness, self-efficacy, intention, attitudes, perceived risk, comprehension, knowledge, acceptance, relevance | N |
| Visser et al.  [68] | 2019 | Single site RCT | Patient Education and Counselling | Does silence speak louder than words? The impact of oncologists' emotion-oriented communication on analogue patients' information recall and emotional stress | Netherlands | 217 | Oncology | Anxiety, health literacy, age | Recall of information | Y |
| von dem Knesebeck et al.  [105] | 2012 | Multi-national factorial experiment | Patient Education and Counselling | Talking about smoking in primary care medical practice—Results of experimental studies from the US, UK and Germany | USA, UK, and Germany | 768 | Smoking | Income, SES, gender/sex, ethnicity | Behavior | N |
| Withers et al.  [73] | 2002 | Multisite RCT | Journal of Psychosomatic Research | A controlled evaluation of an eating disorders primary prevention videotape using the Elaboration Likelihood Model of Persuasion | Australia | 218 | Eating disorders | Disease severity, age, personal relevance | Knowledge, intentions | Y |
