# Supplementary material for: The Role of Recipient Characteristics in Health Video Communication Outcomes: Scoping Review
Source: J Med Internet Res. 2021 Dec 30;23(12):e30962. doi: 10.2196/30962 (PMC8759013; doi:10.2196/30962)
Supplement: Multimedia Appendix 3 [file jmir_v23i12e30962_app3.docx]

**Appendix 3**

| **Characteristics** | **N. papers** | **% papers** |
| --- | --- | --- |
| Age | 33 | 85% |
| Ethnicity/race | 26 | 67% |
| Gender | 25 | 64% |
| Education level | 24 | 62% |
| Income/SES | 13 | 33% |
| Beliefs and attitudes | 12 | 31% |
| Knowledge | 11 | 28% |
| Disease severity | 10 | 26% |
| Previous experience | 10 | 26% |
| Health literacy | 7 | 18% |
| Marital status | 7 | 18% |
| Insurance type | 6 | 15% |
| Preferences | 6 | 15% |
| Risk estimation or perception | 5 | 13% |
| Anxiety | 4 | 10% |
| Values | 4 | 10% |
| Confidence | 3 | 8% |
| Depression | 3 | 8% |
| General health | 3 | 8% |
| Trust in health care | 3 | 8% |
| Trust in information sources | 3 | 8% |
| Current symptoms | 2 | 5% |
| Expectations | 2 | 5% |
| Information | 2 | 5% |
| Perception of knowledge | 2 | 5% |
| Personal relevance | 2 | 5% |
| Self-efficacy | 2 | 5% |
| Ability to process information | 1 | 3% |
| Clinical factors | 1 | 3% |
| Comorbidities | 1 | 3% |
| Culture | 1 | 3% |
| Decisional control preferences | 1 | 3% |
| Efficacy perception | 1 | 3% |
| Emotional factors | 1 | 3% |
| Empathy | 1 | 3% |
| Employment | 1 | 3% |
| Financial comfort | 1 | 3% |
| Health history | 1 | 3% |
| Health locus of control | 1 | 3% |
| Hesitancy | 1 | 3% |
| Hope | 1 | 3% |
| Influential personal factors | 1 | 3% |
| Information and support | 1 | 3% |
| Information priorities | 1 | 3% |
| Involvement | 1 | 3% |
| Living with someone | 1 | 3% |
| Location | 1 | 3% |
| Motivation | 1 | 3% |
| Personality factors | 1 | 3% |
| Psychological distress | 1 | 3% |
| Regulatory focus | 1 | 3% |
| Self-reported adherence | 1 | 3% |
| Sexual orientation | 1 | 3% |
| Social network | 1 | 3% |
| Social norms | 1 | 3% |
| Treatment concerns | 1 | 3% |

Table 1. Frequency of reported characteristics

| **Outcome** | **N. papers** | **% papers** |
| --- | --- | --- |
| Knowledge | 15 | 38% |
| Attitudes | 9 | 23% |
| Behaviour | 9 | 23% |
| Intentions | 8 | 21% |
| Acceptance | 7 | 18% |
| Beliefs | 5 | 13% |
| Choice (of treatment) | 4 | 10% |
| Decisional conflict | 4 | 10% |
| Satisfaction | 4 | 10% |
| Usefulness | 4 | 10% |
| Adherence | 3 | 8% |
| Comprehension | 3 | 8% |
| Emotions | 3 | 8% |
| Information | 3 | 8% |
| Perceived risk | 3 | 8% |
| Reactions | 3 | 8% |
| Recall of information | 3 | 8% |
| Trust | 3 | 8% |
| Confidence | 2 | 5% |
| Decision quality | 2 | 5% |
| Engagement | 2 | 5% |
| Information processing | 2 | 5% |
| Information seeking | 2 | 5% |
| Participation | 2 | 5% |
| Preference (of treatment) | 2 | 5% |
| Quality of communication | 2 | 5% |
| Self-efficacy | 2 | 5% |
| Activation | 1 | 3% |
| Attention | 1 | 3% |
| Awareness | 1 | 3% |
| Benefit perception | 1 | 3% |
| Capabilities | 1 | 3% |
| Compliance | 1 | 3% |
| Decision satisfaction | 1 | 3% |
| Expectations | 1 | 3% |
| Perceived response efficacy | 1 | 3% |
| Perceived self-confidence | 1 | 3% |
| Perception of the prevalence | 1 | 3% |
| Relevance (added value) | 1 | 3% |
| Spreading the message | 1 | 3% |
| Time to treatment | 1 | 3% |
| Uncertainty | 1 | 3% |

Table 2. Frequency of reported outcomes
